# Effects of Isochlorogenic Acid on Ewes Rumen Fermentation, Microbial Diversity and Ewes Immunity of Different Physiological Stages

**DOI:** 10.3390/ani14050715

**Published:** 2024-02-24

**Authors:** Shuyan Li, Xiongxiong Li, Yuzhu Sha, Shuai Qi, Xia Zhang, Huning Wang, Zhengwen Wang, Shengguo Zhao, Ting Jiao

**Affiliations:** 1 College of Pratacultural Science, Gansu Agricultural University, Lanzhou 730070, China; lisy@st.gsau.edu.cn (S.L.); idreamworker@sina.com (S.Q.); 18894012309@139.com (X.Z.); 18419165872@163.com (H.W.); gsauwangzhengwen@163.com (Z.W.); 2 Key Laboratory for Grassland Ecosystem of Ministry of Education, Gansu Agricultural University, Lanzhou 730070, China; 3 College of Animal Science and Technology, Gansu Agricultural University, Lanzhou 730070, China; lxx_gsau@163.com (X.L.); 18894314093@163.com (Y.S.); zhaosg@gsau.edu.cn (S.Z.)

**Keywords:** isochlorogenic acid, ewes, Hu lambs, physiological stages, rumen fermentation, rumen microbial diversity

## Abstract

**Simple Summary:**

In the context of large-scale production, it is necessary to improve ewes health status and reproductive performance to promote newborns survival rate and later fattening performance. Previous studies have indicated that phenolic acids could change the host rumen microflora and inhibit pathogenic bacteria to ensure rumen health and host body health. However, there are few studies on isochlorogenic acid (ICGA) in ruminants. Therefore, based on regulating the rumen environment of breeding ewes during the whole physiological period, the experiment was conducted to study the effects of ICGA on rumen fermentation, microbial diversity and immunity of ewes at estrus, pregnancy and lactation stages. The experimental data obtained showed that adding ICGA could regulate ewes rumen fermentation mode, optimize microbial flora of different physiological stages by increasing *Bacteroidota* relative abundance while reducing *Firmicutes* relative abundance, maintain rumen microbial homeostasis at the pregnancy stage, and increase ewes blood immuneglobulin content, thereby improving ewes health.

**Abstract:**

The effects of isochlorogenic acid (ICGA) on ewes rumen environment, microbial diversity, and immunity at different physiological stages (estrus, pregnancy and lactation) were studied in this experiment. Twenty healthy female Hu lambs of 1.5 months with similar body weight (17.82 ± 0.98 kg) and body condition were selected and randomly divided into two groups: the control group (CON) and the ICGA group (ICGA). The lambs of CON were fed a basal diet, while the lambs of ICGA were supplemented with 0.1% ICGA based on the basal diet. Lambs rumen fermentation characteristics, microbial diversity and immunity at estrus, pregnancy, and lactation stages were determined and analyzed, respectively. The results showed that the rumen pH in CON increased first and then decreased as lambs grew (*p* < 0.05). However, it showed the opposite change in ICGA. The content of ammonia nitrogen (NH_3_-N) showed the highest at estrus stage in both groups, but it was significantly higher in ICGA than that in CON (*p* < 0.05). The Acetic acid/propionic acid (A/P) ratio at estrus stage and the volatile fatty acids (VFAs) at pregnancy stage in ICGA were significantly higher than those of the CON (*p* < 0.05). The 16S rDNA sequencing analysis showed that the Shannon, Chao 1 and ACE indexes of the ICGA were significantly higher than those of the CON both at estrus and lactation stages (*p* < 0.05), while they showed higher at the pregnancy stage in CON (*p* > 0.05). Principal component analysis (PCA) showed that there were significant differences in rumen microorganism structure between CON and ICGA at all physiological stages (*p* < 0.01). At the phylum level, compared with the CON, *Firmicutes* relative abundance of three physiological stages decreased (*p* > 0.05) while *Bacteroidota* increased (*p* > 0.05). The relative abundance of *Synergistota* at estrus stage and *Patescibacteria* at the lactation stage increased significantly too (*p* < 0.05). At the genus level, compared with the CON, the relative abundance of *Prevotella* at three stages showed the highest (*p* > 0.05), while the relative abundance of *Succiniclasticum*, *unclassified_Selenomonadaceae* and *Rikenellaceae_RC9_gut_group* showed different abundances at different physiological stages in ICGA. Compared with the CON, the lambs of the ICGA showed higher blood IgG, IgM, and TNF- α contents at three physiological stages and higher IL-6 contents at pregnancy stage (*p* < 0.05). Conclusion: Adding ICGA could regulate ewes rumen fermentation mode at different physiological stages by increasing rumen NH_3_-N at estrus, VFAs at pregnancy, and the ratio of A/P at lactation. It optimizes rumen microbial flora of different physiological stages by increasing *Bacteroidota* relative abundance while reducing *Firmicutes* relative abundance, maintaining rumen microbial homeostasis at pregnant stage, increasing the number of beneficial bacteria in later lactating and ewes blood immunoglobulins content at three physiological stages.

## 1. Introduction

With the development of urbanization, animal product consumption has undergone a historic turning point in China [1]. People currently prefer mutton to meet their high-quality protein needs [2]. To meet mutton supply, sheep breeding and feeding are gradually transitioning from grazing to large-scale and standardized production [3]. In this context, sheep health status has become one of the focuses, especially ewes, which has directly impacted ewes reproductive performance, growth rate, and lambs’ production performance [4]. Meanwhile, the problems of ewes low immunity, low fecundity, and the survival rate of newborns faced by the animal industry has affected the development of sheep industry seriously [5]. Therefore, it is necessary to improve ewes’ health condition and reproductive performance to promote newborns survival rate and later fattening performance.

*Stevia rebaudiana* was native to the subtropical region of South America [6]. It has been planted widely in both southern and northern regions since it was introduced and cultivated successfully in China in 1977 [7]. In recent years, there have been reports that the stevia extract contained a variety of phenolic acids, such as chlorogenic acid (CGA) and isochlorogenic acid (ICGA), which have biological activities, such as scavenging free radicals, antibacterial and anti-inflammatory, inhibiting tumors, protecting liver and gallbladder, promoting blood circulation and reducing blood pressure, etc. [8,9]. Previous studies indicated that CGA had multiple benefits for animals gastrointestinal health [10]. It can optimize the gastrointestinal flora composition to improve animal intestinal health by increasing short-chain fatty acids (SFAs), promoting intestinal probiotics growth and inhibiting pathogenic bacteria [11,12,13]. ICGA has a similar structure as CGA [14], it has one more caffeoyl group than CGA in structure, and so, has stronger biological activity [15]. In our previous study, we found that adding ICGA to the sheep diet could increase rumen acetic acid (AA), butyric acid (BA), and total volatile fatty acid (TVFA) concentration, so as to regulate the rumen fermentation mode [16].

Rumen is a special digestive and absorptive organ of ruminants [17]. Microorganisms in the rumen not only play a key role in animal digestion and absorption but also play an important role in host immune response and body health [18,19,20]. A large number of studies on cows have shown that ruminal dysbiosis could lead to metabolic changes in rumen and other parts of the digestive tract, causing a large number of microorganisms to release toxins into gastrointestinal lumen, triggering various systemic inflammations and directly affecting the health of next generation [21]. Therefore, from the perspective of ruminant reproductive health, strengthening rumen health is particularly important [22].

Previous studies have shown that ICGA could regulate the rumen fermentation mode. So, we speculate that ICGA may change the host rumen microflora and inhibit pathogenic bacteria to ensure rumen health and host body health. Therefore, the primary aim of this study is to investigate the effects of isochlorogenic acid (ICGA) on rumen fermentation and microbial diversity in ewes across different physiological stages. Our hypothesis is that ICGA can regulate the mode of rumen fermentation and contribute to the optimization of microbial flora, thereby improving the health of Hu sheep.

## 2. Materials and Methods

### 2.1. Experimental Material

The ICGA derived from stevia is purchased from Chenguang Biotechnology Co., Ltd. (Handan, China), with a total acidity greater than 55%.

The 1.5-month-old weaned female lambs of Hu sheep came from Guanghe county, Gansu province, with similar body conditions selected as experimental animals. All of the lambs are in a state of health.

### 2.2. Experimental Design

Twenty 1.5-month-old healthy and homogeneous weaned female lambs with similar body weights (17.82 ± 0.98 kg) were randomly divided into two groups: the control group (CON) and ICGA group (ICGA), 10 lambs in each and housed in one pen (one pen per group) throughout the research. The lambs in CON were fed a basal diet, while the lambs in ICGA were supplemented with 0.1% ICGA based on the basal diet (air-dried basis). The amount of ICGA addition was referred to the results of our previous research of in vitro rumen fermentation [16]. It was weighed and mixed into their diet, stirred thoroughly and evenly before feeding. Lambs in each group were raised in one pen, free feeding and drinking water. After all lambs were fed to sexual maturity (7 months old), embolectomy and estrus synchronization operations were started. Pregnant mare’s serum hormones were injected 10 days later. The embolus was withdrawn after 11 days, then insemination was performed 2 days later, and again 24 h later. Artificial insemination status assessment was conducted 10 days later after the insemination. Ewe pregnancy was checked using B-ultrasound 45 days later, then the successfully pregnant ewe was fed until they gave birth (lactation). Every period the ewes were fed the according diets shown in Table 1. The basal diets of two groups at different physiological stages were formulated according to Agricultural Industry Standard Mutton Sheep Feeding Standard of People’s Republic of China. “Estrus ration” was used during estrus, “Pregnancy ration” during pregnancy, and “Lactation ration” during lactation. The composition and nutritional components of raw materials are shown in Table 1.

### 2.3. Sample Collection and Processing

The ewes rumen fluid and blood samples in each group were collected at estrus, pregnancy (120 days after pregnancy) and lactation (30 days after delivery) stages, respectively. Rumen fluid samples were collected through oral cavity using a rumen tube. Briefly, a flexible PVC tube (2 mm of wall thickness × 6 mm of internal diameter) with holes of 2.5 mm diameter in the 15 cm-probe head (Anscitech Co., Ltd., Wuhan, China) was connected to an electric vacuum pump (7 mbar) and inserted into the ewes rumen via the esophagus to collect the rumen sample. About 75 mL of rumen fluid from each ewe was collected 3 h after morning feeding. The first 5 mL of rumen fluid in each sampling was discarded to remove the potential saliva contamination and the remaining contents were collected. One part was divided into a 50 mL centrifuge tube and stored at −20 °C for fermentation parameters determination; and one part was placed in 5 mL cryotube and stored at −80 °C for rumen microbiota determination. Approximately 10 mL of fasting jugular vein blood in every period were also collected and placed in a common blood collection vessel, centrifuged at 3500 r·min^−1^ for 10 min to obtain the separated serum, then stored at −20 °C for the determination of blood immune indexes.

### 2.4. Rumen Fermentation Index Determination

The pH value was measured by Sartorius acidometer (PB-10). Volatile fatty acids content was determined by the Agilent 7890B meteorological chromatograph. After the rumen fluid sample was pretreated, it was injected into a gas chromatograph to obtain the chromatogram of VFAs sample, then we calculated the content of VFAs using the peak area external standard method [23]. Ammonia nitrogen content was determined by the phenol-sodium hypochlorite colorimetric method. The rumen fluid was centrifuged at 4000 r/min for 10 min, and 2 mL of supernatant was taken into a 15 mL centrifuge tube, 8 mL of 0.2 mol/L hydrochloric acid was added, then shaken well. Then, 2 mL of solution A (0.08 g of sodium nitroso ferricyanide dissolved in 100 mL of 14% sodium salicylate solution) and 2 mL of solution B (2 mL of sodium hypochlorite solution mixed with 100 mL of 0.3 mol/L sodium hydroxide solution) were added in turn, respectively. After shaking and standing for 10 min, the absorbance value was recorded at 700 nm to calculate the ammonia nitrogen content [24].

### 2.5. Analysis of Rumen Microbial Diversity

From DNA extraction to sequencing steps: (1) Microbial total DNA extraction; (2) Target fragment PCR amplification; (3) Amplification products purification and recovery by magnetic beads; (4) Amplification products fluorescence quantification; (5) Sequencing library preparation; (6) High-throughput sequencing; (7) Results analysis, including preliminary screening of the original offline data of high-throughput sequencing according to the sequence quality, the problem samples retesting, then removing the primer fragments of the sequence, discarding the unmatched primers sequence, and performing quality control, denoising, splicing, chimera removal and other steps. Based on valid data, the sequences were clustered into operational taxonomic units (OTUs) with 97% homology, and species annotation analysis was performed using OTUs sequences and the RDP Classifier database. According to the results of species annotation, alpha diversity and beta diversity were calculated, and the differences between groups were compared to reveal the differences of the same flora under each treatment. The diversity index was calculated based on the OTU level. Beta diversity analysis (PCA) was used to analyze the differences in community structure among different populations.

### 2.6. Blood Immune Index Determination

Serum immunoglobulin A (IgA), immunoglobulin G (IgG), immunoglobulin M (IgM), interleukin-1β (IL-1β), interleukin-6 (IL-6) and Tumor necrosis factor α (TNF-α) were measured using commercial ELISA kits (Nanjing Jiancheng Biotech, Nanjing, Jiangsu, China) [25].

### 2.7. Statistical Analysis

Excel 2010 was used to sort out the experimental data and the independent sample *t*-test in SPSS 23.0 software was used to analyze the significance between different treatments at the same physiological stage. *p* < 0.05 indicated the difference was significant, while *p* < 0.01 showed extremely significant.

## 3. Results

### 3.1. Effects of ICGA on Ewes Rumen Fermentation at Different Physiological Stages

With the advancement of ewes physiological periods, the ewes rumen pH increased at pregnancy and then decreased at lactation in CON, while the content of NH_3_-N and VFAs decreased at pregnancy and then increased at lactation, while in ICGA, the ewes rumen NH_3_-N content decreased first and then increased, but the pH and VFAs content showed the opposite change (Table 2). Compared with the CON, the rumen propionic acid (PA), butyric acid (BA) and TVFA content decreased significantly, and the A/P ratio and NH_3_-N content increased significantly (*p* < 0.05) at estrus stage, while acetic acid (AA), PA, isobutyric acid (IBA), BA, isovaleric acid (IVA), valeric acid (VA) and TVFA increased (*p* < 0.05) at pregnancy stage in ICGA. There were no significant differences in rumen fermentation parameters between CON and ICGA at lactation stage (*p* > 0.05) (Table 2).

### 3.2. Effects of ICGA on Ewes Rumen Microbial Diversity at Different Physiological Stages

#### 3.2.1. Effect on the Number of Operational Taxonomic Units (OUT)

**Figure 1:**
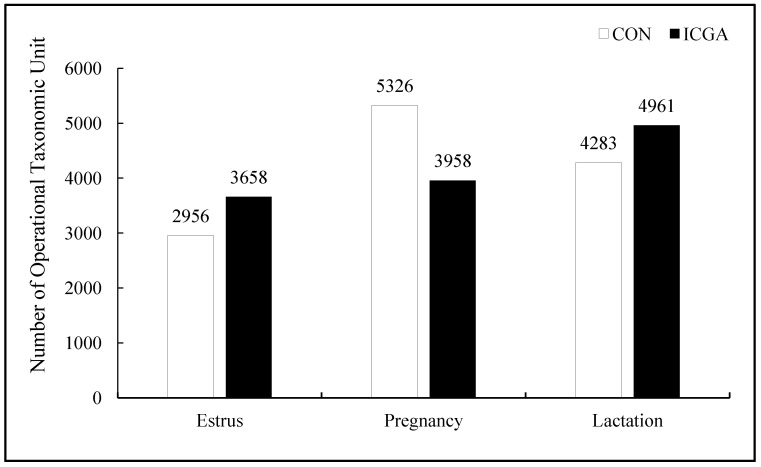
Operational Taxonomic Unit number distribution map.

**Figure 2:**
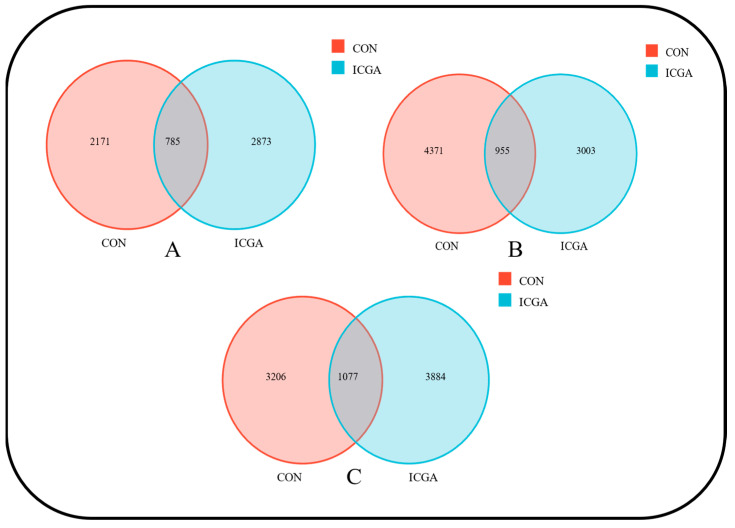
Venn diagram for OTU distribution of rumen microorganisms. (**A**): Estrus; (**B**): Pregnancy; (**C**): Lactation.

A total of 18,302 OTUs were obtained in this sequencing. The number of OTUs were higher at estrus and lactation stage while they were lower at pregnancy stage in ICGA than those in CON (Figure 1). There were 2171 unique OTUs in CON and 2873 unique OTUs in ICGA, with a total of 785 OTUs at estrus stage (Figure 2A), 4371 unique OTUs in CON and 3303 unique OTUs in ICGA, with a total of 955 OTUs at pregnancy stage (Figure 2B) and 3206 unique OTUs in CON and 3884 unique OTUs in ICGA, with a total of 1077 OTUs at lactation stage (Figure 2C).

#### 3.2.2. Effect on Dilution Curve

**Figure 3:**
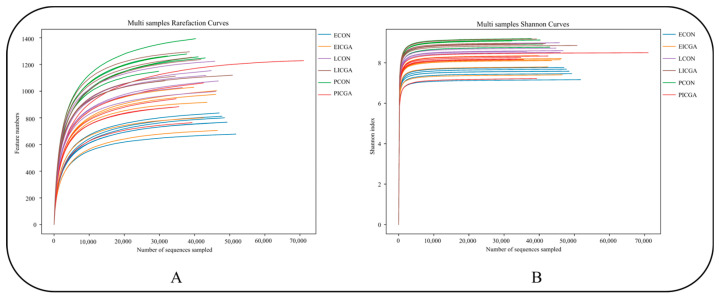
Sample dilution curve. (**A**): OTU number dilution curve; (**B**): Shannon index dilution curve.

The sample dilution curves of ewes three physiological stages in both groups showed a trend of rising first and then leveling off (Figure 3), indicating that the sequencing depth was sufficient to cover all species in the sample, and the sequencing results were reliable.

#### 3.2.3. Effect on Alpha Diversity

**Figure 4:**
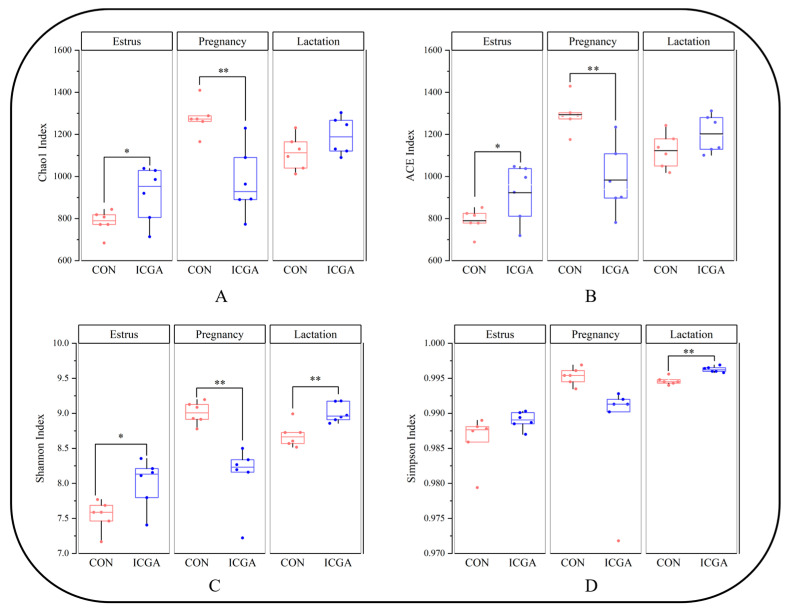
Alpha diversity index of rumen microbes. (**A**): Chao 1 Index; (**B**): ACE Index; (**C**): Shannon Index; (**D**): Simpson Index. * *p* < 0.05, ** *p* < 0.01.

The Shannon, Chao 1, and ACE indexes of the ICGA at estrus stage were significantly higher and those at the pregnancy stage were significantly lower than those of the CON group (*p* < 0.05). The Shannon and Simpson indexes of the ICGA were significantly higher than those of the CON at lactation stage (*p* < 0.05) (Figure 4).

#### 3.2.4. Effect on Beta Diversity

**Figure 5:**
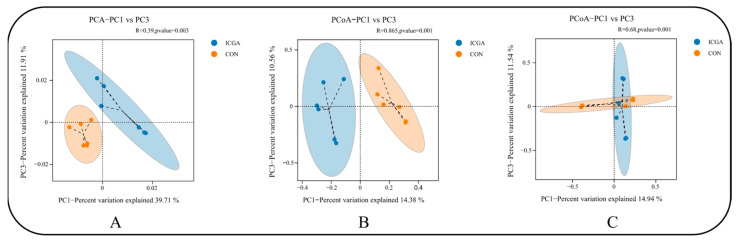
PCA of rumen microflora. (**A**): Estrus; (**B**): Pregnancy; (**C**): Lactation.

There were significant differences in rumen microflora between CON and ICGA at estrus (Figure 5A), pregnancy (Figure 5B) and lactation (Figure 5C) stage (*p* < 0.01).

#### 3.2.5. Effect on Bacterial Composition and Structure at the Phylum Level

**Figure 6:**
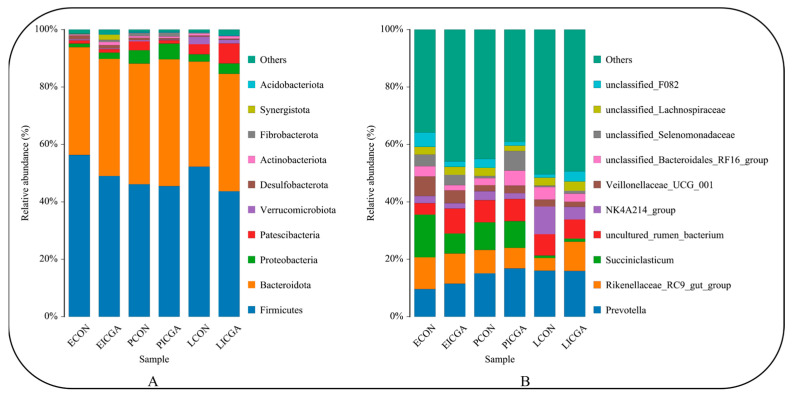
The relative abundance of phylum (**A**) and genus (**B**) of rumen microbiota Top 10.

At the phylum classification level, the top 10 species in the relative abundance of rumen microbial communities at three physiological stages of two groups were counted (Figure 6A) (Table 3). With the advancement of the physiological periods, the relative abundance of *Firmicutes* in CON decreased first and then increased, while that in ICGA decreased. The relative abundance of *Bacteroidota* in both groups increased first and then decreased. The relative abundance of *Firmicutes* in both groups were highest at three physiological stages, followed by *Bacteroidota*. Compared with the CON, the relative abundance of *Firmicutes* in ICGA was lower (*p* > 0.05), and the relative abundance of *Bacteroidota* and *Proteobacteria* were higher than those in CON (*p* > 0.05). The relative abundance of *Synergistota* in ICGA was significantly higher than that in CON at the estrus stage (*p* < 0.05), but the relative abundance of *Patescibacteria* in ICGA was significantly higher than that in CON at lactation stage (*p* < 0.05).

#### 3.2.6. Effect on Bacterial Composition and Structure at the Genus Level

At the genus classification level, the top 10 species in the relative abundance of rumen microbial communities in CON and ICGA at three physiological stages were counted (Figure 6B) (Table 4). With the advancement of the physiological periods, the relative abundance of *Prevotella* in CON increased gradually, while the relative abundance of *Rikenellaceae_RC9_gut_group* and *Succiniclasticum* decreased. The relative abundance of *Prevotella* and *Succiniclasticum* in ICGA increased first and then decreased, and the relative abundance of *Rikenellaceae_RC9_gut_group* decreased first and then increased. Compared with the CON, the relative abundance of *Prevotella* in ICGA was higher at three physiological stages (*p* > 0.05), the relative abundance of *Rikenellaceae_RC9_gut_group* in ICGA at lactation stage was significantly higher (*p* < 0.05), and the relative abundance of *NK4A214_group* was significantly lower (*p* < 0.05). The relative abundance of *unclassified_Selenomonadaceae* was significantly higher at the pregnancy stage (*p* < 0.05) while the relative abundance of *Succiniclasticum* and *unclassified_F082* was significantly lower at the estrus stage in ICGA than those in CON (*p* < 0.05).

#### 3.2.7. Effect on LEfSe Difference

**Figure 7:**
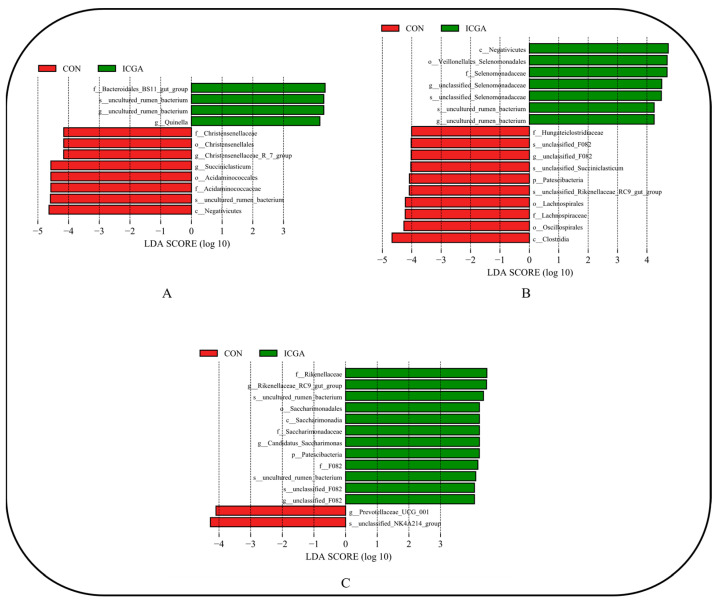
LDA value distribution histogram. (**A**): Estrus; (**B**): Pregnancy; (**C**): Lactation.

LEfSe difference analysis was performed on rumen microorganisms of two groups at three physiological stages at different classification levels (Figure 7). At the estrus stage (Figure 7A), the ewes in CON had eight species groups with significant differences. They were mainly *Negativicutes*, *uncultured_rumen_bacterium*, *Acidaminococcales*, *Acidaminococcaeae*, *Succiniclasticum*, and *Christensenellaceae_R7_group*. There were four significantly different microbial groups in ICGA, mainly *Bacteroidales_BS11_gut_group*, *uncultured_rumen_bacterium* and *Quinella*. At the pregnancy stage (Figure 7B), there were ten significantly different species groups in CON, mainly including *Clostridia*, *Oscillospirales*, *Lachnospiraceae* and *unclassified_Rikenellaceae_RC9_gut_group*. There were seven significantly different microbial groups in ICGA, mainly for *Negativicutes*, *Veillonellales-selenomonadales*, *unclassified_Selenomonadaceae*, and *uncultured_rumen_bacterium*. At the lactation stage (Figure 7C), there were two significantly different species groups in CON, which were *unclassified_NK4A214-group* and *Prevotellaceae_UCG_001*, respectively. There were twelve significantly different microbial groups in ICGA, mainly *Rikenellaceae*, *Rikenellaceae_RC9_gut_group* and *uncultured_rumen_bacterium*.

### 3.3. Effects of ICGA on Ewes Blood Immune Index at Different Physiological Stages

**Figure 8:**
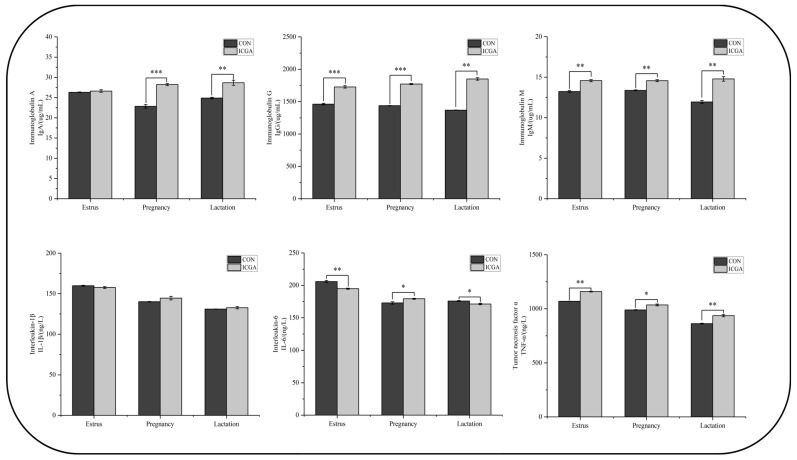
Effects of ICGA on ewes blood immune index at different physiological stages. * *p* < 0.05, ** *p* < 0.01, *** *p* < 0.001.

Compared with the CON, the ewes of ICGA showed higher serum IgG, IgM, TNF- α contents at three physiological stages (*p* < 0.05), while lower serum IL-6 contents at the estrus and lactation stages, but higher IL-6 at the pregnancy stage (*p* < 0.05) (Figure 8).

## 4. Discussion

### 4.1. Effects of ICGA on Ewes Rumen Fermentation at Different Physiological Stages

Ruminant rumen is a relatively stable anaerobic fermentation tank. NH_3_-N, pH, and VFAs are the main internal environmental indicators for rumen fermentation [26]. In this experiment, the rumen pH is within the normal range of 6.3 and 7.0 at three physiological stages in both groups, meaning that there was no adverse effect on ewes rumen fermentation by ICGA. The rumen NH_3_-N content had a significant increase at estrus in ICGA, which was beneficial to synthesize more microbial protein (MCP) to provide large amounts of protein for body, so improve ovulation rate and conception rate of ewes at estrus stage [27]. Weston pointed out the TVFA content decreased with the prolongation of pregnancy time [28], which may be due to ewes absorbing VFAs rapidly by rumen wall to need higher energy demand because of the rapid development of pregnant embryos, thus reduce the rumen VFAs concentration of pregnant ewes. Adding ICGA may stimulate the rumen microorganisms (*unclassified_Selenomonadaceae*) of pregnant ewes to produce VFAs, providing sufficient energy for pregnant ewes, which is beneficial to the health of ewes and embryos [29]. Due to the heavy burden and nutrient consumption at the pregnancy stage, the feed intake of lactating ewes increased, and the rumen NH_3_-N and VFAs content also increased [30,31] However, the NH_3_-N and VFAs contents showed the opposite in ICGA. Adding ICGA may accelerate the absorption of NH_3_-N and VFAs in the rumen wall so that the ewes rumen NH_3_-N and VFAs contents decrease at the lactation stage in ICGA. Liu Yongjun pointed out the high-yield group of dairy cows had a stronger absorption capacity for VFAs produced by rumen fermentation [32]. In this experiment, the A/P of the ICGA was higher than that of the CON at the estrus and lactation stages and was significantly lower than that of the CON at the pregnancy stage. AA is an important precursor for synthesis of milk fat, PA is the precursor of body fat synthesis, and the A/P ratio is significantly positively correlated with milk fat rate [33,34]. Adding ICGA could promote the synthesis of body fat in estrus ewes and accumulate energy for pregnancy, promote synthesis of glucose from propionic acid to provide more energy for pregnant ewes and synthesis of milk fat in lactating ewes to increase the milk fat rate of milk.

### 4.2. Effects of ICGA on Ewes Rumen Microbial Diversity at Different Physiological Stages

Through sequencing data analysis, it was found that the diversity dilution curve of rumen flora increased first and then tended to be gentle, indicating that the depth of this sequencing was sufficient and the sequencing results were reliable. The rumen microbial community is dynamic and often changes with diet, host, physiological state and environment [35]. In ICGA, the rumen microorganisms richness and diversity at estrus and lactation stages were higher than those of the CON while lower than those of the CON at pregnancy stage. It may be due to the stress response during pregnancy that the rumen fluid flora in CON was disordered, resulting in an increase of rumen species richness in CON during pregnancy. Guo pointed out the rumen flora diversity of ewes increased at 120 days of pregnancy [36]. It is speculated that adding ICGA to the diet may maintain the rumen microbial homeostasis of pregnant ewes and alleviate pregnant ewes stress response. Through Beta diversity analysis of samples, we found that the sample composition of CON and ICGA at different physiological stages was significantly separated, and there were significant differences in rumen microflora. So, adding ICGA could change the ewes rumen microbial flora structure at estrus, pregnancy and lactation stages.

The analysis of rumen microflora composition and structure at the phylum level showed that the dominant phyla in CON and ICGA were *Firmicutes* and *Bacteroidota* at three physiological stages, and the dominant phyla distribution was similar to results of other studies [37]. In this experiment, the relative abundance of *Firmicutes* in ICGA was lower than that in CON, while the relative abundance of *Bacteroidota* was higher than that in CON. High abundance of *Bacteroidota* usually has anti-inflammatory effects [38], showing ICGA may exert anti-inflammatory effects by reducing *Firmicutes* abundance and increasing *Bacteroidota* abundance, so increase the ratio of *Bacteroidota* to *Firmicutes*, the same as other results [39], which can reduce fat ewes deposition, thereby accelerating material circulation and energy flow to be benefit to maintain ewes health [40]. It is also indicated that ICGA can promote the protein and non-structural carbohydrates degradation and feed absorption and transformation in rumen by reducing *Firmicutes* abundance and increasing *Bacteroidota* abundance [41]. The relative abundance of *Synergistota* at estrus stage and the relative abundance of *Patescibacteria* at lactation stage in ICGA both were significantly increased. Studies showed that *Synergistota* isolated from ruminant foods could use arginine and histidine as substrates to degrade toxins in diet, while *Patescibacteria* has a strong ability to adapt to adverse environments [42,43]. It can be suggested that ICGA may improve ewes stress resistance on adverse environments to help host animals survive well.

The composition and structure analysis of rumen microbial flora at the genus level showed that the relative abundance of *Prevotella* in ICGA was higher than that in CON at three physiological stages. *Prevotella* is the dominant genus in mature rumen and the most abundant and common group of rumen microorganisms [44]. ICGA may promote rumen microflora maturation [45]. In this experiment, the relative abundance of *unclassified_Selenomonadaceae* in ICGA at pregnancy stage was significantly higher than that in CON, which could promote VFAs production in pregnant ewes rumen [46]. It was consistent with the status of pregnant ewes rumen fermentation. Studies showed that the *Rikenellaceae_RC9_gut_group* could promote the digestion and absorption of carbohydrates in intestine and protect mucosal barrier function by increasing butyrate levels [47,48]. Just in time, the relative abundance of *Rikenellaceae_RC9_gut_group* in ICGA was significantly higher than that in CON in the experiment. ICGA could improve lactating ewes rumen health by increasing rumen beneficial bacteria amounts and promoting rumen VFAs absorption and utilization of lactating ewes., The relative abundance of *Succiniclasticum* at the estrus stage in ICGA was significantly lower than that in CON in the experiment. *Succiniclasticum* played an important role in PA production, which may be the reason for the decrease of rumen PA concentration at estrus stage in ICGA [49].

LEfSe difference analysis showed that *Negativicutes* species were significantly enriched in CON at estrus, and *Bacteroidales_BS11_gut_group* and *Quinella* were significantly enriched in ICGA. The studies showed that the increase in *Quinella* abundance was related to a decrease in methane production [50]. It was speculated that ICGA not only promoted the ewes protein and soluble carbohydrates degradation at estrus but also inhibited methane production. At pregnancy, the species significantly enriched in CON were mostly *Clostridia*; *Negativicutes* species in ICGA were significantly enriched. Both *Clostridia* and *Negativicutes* belong to *Firmicutes*, which could promote fiber degradation to provide energy for pregnant ewes. At lactation, *Prevotellaceae_UCG_001* was significantly enriched in CON, and *Rikenellaceae* and *Rikenellaceae_RC9_gut_group* were significantly enriched in ICGA. ICGA could increase rumen beneficial bacteria of postpartum lactating ewes, promote ewes carbohydrates digestion and absorption at lactating, so maintain lactating ewes rumen health.

### 4.3. Effects of ICGA on Ewes Blood Immune Index at Different Physiological Stages

Immunoglobulin is a type of large molecular protein that is rapidly produced after being stimulated by foreign pathogens and can combine with antigens to enhance the body’s resistance to stress, thereby promoting animal overall health [51]. In this experiment, the ewes serum IgG, IgM, and TNF-α contents increased significantly at three physiological stages in ICGA, showing that ICGA can increase ewes serum immunoglobulin contents. Once external pathogens invade the body, they quickly bind with complement, dissolve bacteria, and prevent or reduce the occurrence of stillbirth, miscarriage, and other phenomena [52]. It can also promote the proliferation of immune cells, improve immune ability, and ewes health status [53]. In this experiment, the blood IL-6 contents in ICGA ewes was significantly higher than that of the CON at pregnancy stage. IL-6 belongs to the inflammatory cytokine family and is one of the essential components in the complex cytokine network in animal. Low IL-6 value may lead to trophoblast invasion into the surface and hinder placental vascular formation, resulting in placental trophoblast ischemia [54]. Researchers found that IL-6 mRNA levels were significantly decreased in preeclamptic placentas before the development of gestational hypertension syndrome [55]. So, adding ICGA can prevent gestational hypertension syndrome in pregnant ewes and maintain maternal pregnancy health.

## 5. Conclusions

ICGA could regulate ewes rumen fermentation mode at different physiological stages by increasing rumen NH_3_-N content at the estrus stage, the VFAs content at the pregnancy stage, and the ratio of A/P at the lactation stage, optimizing rumen microbial flora of different physiological stages by increasing *Bacteroidota* relative abundance while reducing *Firmicutes* relative abundance. This maintains the rumen microbial homeostasis at the pregnancy stage, and increases the number of beneficial bacteria in later lactating and ewes blood immunoglobulins content at three physiological stages, therefore reducing the use of antibiotics and achieving healthy breeding purposes.

## Figures and Tables

**Table 1:** Diet composition and nutritional levels of ewes at different physiological stages (air-dried basis).

Physiological Period		Estrus (7.5 Months Old)	Pregnancy (7.5–12.5 Months Old)	Lactation (12.5–14.5 Months Old)
Formula composition/%	Whole plant silage corn	20	10	22
	Wheat straw	25	25	10
	Alfalfa	25	30	33
	Corn grain	13	15	15
	Bran	2	5	5
	Sesame cake	8	7	7
	Cottonseed meal	3	4	4
	Baking soda	0.5	0.5	0.5
	Calcium hydrogen phosphate	1	1	1
	Salt	0.5	0.5	0.5
	Premix ^1^	2	2	2
	Total	100	100	100
Nutritional level	Dry matter, DM (%)	80.19	85.91	82.85
	Digestible energy, DE/(MJ kg ^−1^)	11.01	11.53	13.03
	Metabolizable energy, ME/(MJ kg ^−1^)	9.07	8.85	9.12
	Calcium, Ca (%)	1.94	1.07	1.08
	Phosphorus, P (%)	0.71	0.54	0.58
	Crude protein, CP (%)	12.89	13.76	13.07

^1^ The premix provided the following per kg of the diet: Cu 5.40 mg, Fe 19.80 mg, Zn 9.00 mg, Mn 14.40 mg, Se 0.11 mg, I 0.18 mg, Co 0.07 mg, VA 7200 IU, VD 1440 IU, VE 500 IU; Metabolic energy and digestible energy were calculated, and other values are measured.

**Table 2:** Effects of ICGA on ewes rumen fermentation at different physiological stages.

Index	Estrus			Pregnancy			Lactation		
	CON	ICGA	*p* Value	CON	ICGA	*p* Value	CON	ICGA	*p* Value
pH	6.55 ± 0.05 ^b^	6.91 ± 0.02 ^a^	0.002	6.73 ± 0.13 ^a^	6.28 ± 0.02 ^b^	0.025	6.52 ± 0.15 ^a^	6.93 ± 0.04 ^a^	0.053
Ammonia nitrogen (mg dL^−1^)	8.62 ± 0.19 ^b^	10.50 ± 0.60 ^a^	0.040	4.79 ± 0.78 ^a^	4.16 ± 0.20 ^a^	0.481	8.10 ± 0.94 ^a^	4.73 ± 0.25 ^a^	0.061
Acetic acid (mmol L^−1^)	49.39 ± 3.49 ^a^	44.18 ± 1.08 ^a^	0.227	26.14 ± 0.46 ^b^	45.63 ± 2.14 ^a^	0.009	45.30 ± 1.91 ^a^	38.33 ± 3.72 ^a^	0.172
Propionic acid (mmol L^−1^)	12.91 ± 0.85 ^a^	9.36 ± 0.46 ^b^	0.021	6.64 ± 0.67 ^b^	18.99 ± 1.37 ^a^	0.001	15.00 ± 1.15 ^a^	10.84 ± 1.07 ^a^	0.057
Isobutyric acid (mmol L^−1^)	0.59 ± 0.04 ^a^	0.66 ± 0.02 ^a^	0.214	0.26 ± 0.02 ^b^	0.41 ± 0.03 ^a^	0.011	0.55 ± 0.11 ^a^	0.43 ± 0.01 ^a^	0.405
Butyric acid (mmol L^−1^)	7.99 ± 0.46 ^a^	4.58 ± 0.20 ^b^	0.002	3.31 ± 0.11 ^b^	7.26 ± 0.39 ^a^	0.001	6.58 ± 0.79 ^a^	5.49 ± 0.40 ^a^	0.284
Isovaleric acid (mmol L^−1^)	0.93 ± 0.06 ^a^	0.95 ± 0.10 ^a^	0.872	0.32 ± 0.00 ^b^	0.64 ± 0.02 ^a^	0.003	0.88 ± 0.18 ^a^	0.78 ± 0.04 ^a^	0.638
Valeric acid (mmol L^−1^)	0.52 ± 0.01 ^a^	0.48 ± 0.03 ^a^	0.365	0.27 ± 0.02 ^b^	0.57 ± 0.05 ^a^	0.006	0.64 ± 0.06 ^a^	0.61 ± 0.02 ^a^	0.715
Acetic acid/Propionic acid	3.82 ± 0.02 ^b^	4.74 ± 0.16 ^a^	0.029	4.00 ± 0.34 ^a^	2.42 ± 0.12 ^b^	0.012	3.04 ± 0.11 ^a^	3.55 ± 0.21 ^a^	0.100
Total volatile fatty acids (mmol L^−1^))	71.45 ± 3.69 ^a^	59.1 ± 2.21 ^b^	0.045	36.88 ± 1.07 ^b^	78.31 ± 4.78 ^a^	0.001	67.17 ± 3.69 ^a^	56.39 ± 5.21 ^a^	0.167

^a,b^ Values with different treatments in the same physiological stage are significantly different (*p* < 0.05).

**Table 3:** Rumen microbial relative abundance at phylum level Top 10 (%).

Phylum	Estrus			Pregnancy			Lactation		
	CON	ICGA	*p* Value	CON	ICGA	*p* Value	CON	ICGA	*p* Value
*Firmicutes*	56.05 ± 0.04	48.86 ± 0.02	0.99	46.42 ± 0.04	45.78 ± 0.02	1.00	51.80 ± 0.03	42.78 ± 0.04	0.93
*Bacteroidota*	37.79 ± 0.04	40.78 ± 0.02	1.00	42.13 ± 0.02	42.86 ± 0.02	1.00	36.92 ± 0.02	41.78 ± 0.05	0.99
*Proteobacteria*	1.26 ± 0.00	2.25 ± 0.01	1.00	4.78 ± 0.01	5.68 ± 0.02	1.00	2.60 ± 0.00	3.70 ± 0.00	1.00
*Patescibacteria*	1.09 ± 0.00	1.22 ± 0.00	1.00	3.11 ± 0.00	0.92 ± 0.00	0.06	3.51 ± 0.00	6.92 ± 0.01	0.00
*Verrucomicrobiota*	0.41 ± 0.00	0.28 ± 0.00	1.00	0.44 ± 0.00	0.27 ± 0.00	1.00	2.63 ± 0.01	1.26 ± 0.00	0.48
*Desulfobacterota*	1.28 ± 0.00	1.13 ± 0.00	1.00	0.80 ± 0.00	0.48 ± 0.00	0.99	0.38 ± 0.00	0.33 ± 0.00	1.00
*Actinobacteriota*	0.27 ± 0.00	1.13 ± 0.01	0.55	0.63 ± 0.00	0.66 ± 0.00	1.00	0.67 ± 0.00	0.87 ± 0.00	1.00
*Fibrobacterota*	0.35 ± 0.00	0.72 ± 0.00	1.00	1.16 ± 0.01	1.64 ± 0.01	1.00	0.38 ± 0.00	0.11 ± 0.00	1.00
*Synergistota*	0.06 ± 0.00	1.88 ± 0.01	0.00	0.04 ± 0.00	0.03 ± 0.00	1.00	0.03 ± 0.00	0.05 ± 0.00	1.00
*Acidobacteriota*	0.01 ± 0.00	0.35 ± 0.00	0.41	0.34 ± 0.00	0.31 ± 0.00	1.00	0.26 ± 0.00	0.40 ± 0.00	1.00

**Table 4:** Rumen microbial relative abundance at genus levels Top10 (%).

Genus	Estrus			Pregnancy			Lactation		
	CON	ICGA	*p* Value	CON	ICGA	*p* Value	CON	ICGA	*p* Value
*Prevotella*	9.67 ± 0.01	11.49 ± 0.01	1.00	14.97 ± 0.01	16.78 ± 0.01	1.00	16.09 ± 0.01	16.44 ± 0.03	1.00
*Rikenellaceae_RC9_gu-t_group*	11.16 ± 0.01	10.44 ± 0.01	1.00	8.13 ± 0.01	6.89 ± 0.00	1.00	4.54 ± 0.01	10.36 ± 0.01	0.00
*Succiniclasticum*	14.80 ± 0.01	6.98 ± 0.01	0.00	9.45 ± 0.01	9.78 ± 0.02	1.00	0.88 ± 0.00	1.11 ± 0.00	1.00
*uncultured_rumen_bac-terium*	4.00 ± 0.01	8.70 ± 0.02	0.23	7.90 ± 0.01	7.19 ± 0.02	1.00	7.53 ± 0.01	6.76 ± 0.01	1.00
*NK4A214_group*	2.46 ± 0.00	1.89 ± 0.00	1.00	3.08 ± 0.00	2.05 ± 0.00	1.00	9.54 ± 0.02	4.32 ± 0.00	0.00
*Veillonellaceae_UCG_001*	6.76 ± 0.01	4.45 ± 0.01	0.27	2.08 ± 0.00	2.95 ± 0.01	1.00	2.4 ± 0.01	1.81 ± 0.00	1.00
*unclassified_Bacteroid-ales_RF16_group*	3.56 ± 0.01	1.81 ± 0.01	0.91	2.40 ± 0.01	5.59 ± 0.02	0.09	4.45 ± 0.01	2.79 ± 0.00	0.94
*unclassified_Selenom-onadaceae*	4.11 ± 0.01	3.57 ± 0.01	1.00	0.72 ± 0.00	6.87 ± 0.04	0.02	0.52 ± 0.00	0.94 ± 0.00	1.00
*unclassified_Lachnospi-raceae*	2.75 ± 0.00	2.77 ± 0.01	1.00	2.87 ± 0.00	2.00 ± 0.00	1.00	2.77 ± 0.01	3.2 ± 0.00	1.00
*unclassified_F082*	5.09 ± 0.01	1.93 ± 0.01	0.02	3.05 ± 0.00	1.14 ± 0.00	0.56	1.15 ± 0.00	3.56 ± 0.00	0.19

## Data Availability

The data presented in this study are available on request from the corresponding author.
